# Longitudinal Neurostimulation in Older Adults Improves Working Memory

**DOI:** 10.1371/journal.pone.0121904

**Published:** 2015-04-07

**Authors:** Kevin T. Jones, Jaclyn A. Stephens, Mahtab Alam, Marom Bikson, Marian E. Berryhill

**Affiliations:** 1 Memory and Brain Laboratory, Department of Psychology, University of Nevada, Reno, Nevada, United States of America; 2 Cognitive Neuropsychology Lab, Department of Neurology, Georgetown University Medical Center, Washington, District of Columbia, United States of America; 3 Department of Biomedical Engineering, The City College of New York, New York, New York, United States of America; University Medical Center Goettingen, GERMANY

## Abstract

An increasing concern affecting a growing aging population is working memory (WM) decline. Consequently, there is great interest in improving or stabilizing WM, which drives expanded use of brain training exercises. Such regimens generally result in temporary WM benefits to the trained tasks but minimal transfer of benefit to untrained tasks. Pairing training with neurostimulation may stabilize or improve WM performance by enhancing plasticity and strengthening WM-related cortical networks. We tested this possibility in healthy older adults. Participants received 10 sessions of sham (control) or active (anodal, 1.5 mA) tDCS to the right prefrontal, parietal, or prefrontal/parietal (alternating) cortices. After ten minutes of sham or active tDCS, participants performed verbal and visual WM training tasks. On the first, tenth, and follow-up sessions, participants performed transfer WM tasks including the spatial 2-back, Stroop, and digit span tasks. The results demonstrated that all groups benefited from WM training, as expected. However, at follow-up 1-month after training ended, only the participants in the active tDCS groups maintained significant improvement. Importantly, this pattern was observed for both trained *and* transfer tasks. These results demonstrate that tDCS-linked WM training can provide long-term benefits in maintaining cognitive training benefits and extending them to untrained tasks.

## Introduction

Working memory (WM) serves as the mental workspace permitting the maintenance and manipulation of information over short delays. Unfortunately, aging impairs WM—a worrisome and frustrating development beginning in our mid-20s [1]. This decline is likely caused by age-related cortical volume loss, particularly in frontoparietal regions engaged in WM [2, 3]. Furthermore, with age, these regions change their functional activation patterns during working memory tasks, showing greater bilateral recruitment at lower task demands (reviewed in [4–6]). This may reflect recruitment of additional frontal resources to maintain performance [7].

Age-related decline in WM affects everyone as we age, and WM function underlies complex cognition [1, 8–10]. Therefore, there is great incentive to develop interventions that stabilize and restore WM performance. Some lifestyle modifications show correlational benefits. For example, cognition improves after increasing physical exercise [11–15] or socialization [16], or adopting a Mediterranean diet [17]. Empirical findings show that WM can benefit from cognitive training (reviewed in [18–21]). It is noteworthy, however, that the ultimate goal of WM training is to produce generalizable improvements rather than to produce prodigies at specific WM tasks [22]. Ideally, this *transfer* represents improved cognitive functioning during WM task performance. In older adults, WM training studies show improved performance on the trained tasks [13, 15, 23–37]. Several report significant transfer to untrained tasks [24, 26, 29], whereas others fail to observe significant transfer [38, 39].

One way to target frontoparietal networks engaged in WM is through neurostimulation, such as transcranial direct current stimulation (tDCS). TDCS has practical translational potential because it is safe [40], well-tolerated [41], and relatively affordable compared to other techniques such as transcranial magnetic stimulation (TMS). TDCS involves applying small amounts (1–2 mA) of electric current to scalp electrodes to modulate the excitability of underlying neural populations [42–46]. Importantly, tDCS is used in patient populations and has shown benefits in treating depression [47, 48], reducing episodic memory deficits in Alzheimer’s [49] and Parkinson’s diseases [50], ameliorating aphasia [51–53], and improving post-stroke motor function [54–56]. Apart from special populations, healthy older adults show tDCS-linked benefits including improved motor function [57, 58], proper name recall [59], and decision-making [60]. Of greatest relevance here, tDCS reliably improves WM in healthy adults [61–73], as well as those with Parkinson’s [50], Alzheimer’s disease [74–76], and stroke [77]. Finally, emerging data show persistent benefits for a month or even a year in various cognitive tasks [78].

To date, tDCS has only rarely been paired with WM training in healthy older adults [79]. This large and growing population will certainly increase the demand for interventions, which can improve WM. Given the research showing that transfer effects are modest or non-existent following behavioral WM training, neuromodulatory techniques may provide the added neural ‘boost’ to enhance and prolong transfer effects. The current study tested whether longitudinal right frontoparietal tDCS-linked WM training would improve WM *and* show significant transfer to untrained tasks. We predicted that active frontoparietal tDCS would improve WM performance on trained and transfer tasks in participants who received active tDCS rather than sham stimulation. The pattern of enhanced frontal activations in the healthy aging suggested that prefrontal stimulation might provide optimal benefits compared to parietal stimulation.

## Materials and Methods

To investigate the *longitudinal* effects of tDCS-linked WM training in a healthy aging population, we tested participants in a tDCS-linked WM training paradigm in which they completed 10 training sessions consisting of 10 minutes of sham (control) or active anodal tDCS. Participants returned for follow-up testing 1-month after training ended. The WM training tasks included verbal and visuospatial tasks and the Operation Span (OSpan) task [80]. To assess transfer, participants completed a set of transfer tasks during the first, tenth, and follow-up sessions.

### Participants

72 neurotypical right-handed older adults (mean (M) age 64.38, standard deviation (SD) 5.08, 49 females (age range 55–73) participated. All participants scored a 25 or higher on the Mini-Mental Status Examination (MMSE). We screened all participants and excluded people with pacemakers, a history of neurological or psychiatric disease, or those on medications modulating brain excitability (e.g. neuroleptic, hypnotic, antidepressant). We randomly assigned participants to one of 4 groups (control (sham), PFC, PPC, PFC/PPC alternating) so that each group had 18 participants. Groups showed no significant differences as a function of age (p = .98; control 64.33 (5.24), PFC 63.94 (4.30), PPC 64.72 (5.72), PFC/PPC 65.50 (5.34), education (p = .96; control 16.72 (2.29), PFC 16.50 (2.79), PPC 16.94 (3.57), PFC/PPC 16.94 (2.84), or MMSE score (p = .68; control 28.61 (1.50), PFC 28.22 (1.56), PPC 28.33 (1.50), PFC/PPC 28.77 (1.52). Participants signed informed consent documents, the University of Nevada Institutional Review Board approved all procedures, and they received $15/hour.

### Behavioral Measures and Training Sequence

Participants completed 10 consecutive weekday sessions (2 weeks: Monday-Friday) and a follow-up session 1-month after the 10^th^ session. During the first session, prior to stimulation, participants completed the MMSE [81], forward and backward digit span [82], color-word Stroop task [83], and spatial 2-back task [84]. The digit span, Stroop and 2-back tasks were considered untrained *transfer* tasks because they were only completed on the 1^st^, 10^th^ and follow-up sessions. During sessions 1–10, participants received tDCS (parameters below) during which they practiced the visuospatial WM task. After stimulation, the participants completed the visuospatial WM tasks and the Automated Operation Span [80]. The 1^st^, 10^th^ and follow-up sessions, lasted 75–90 minutes; sessions 2–9 lasted ~60 minutes. Participants sat 57 cm from the stimulus monitor during computerized tasks.

We picked a series of difficult WM tasks for training. These tasks were selected because they tap in to core WM capabilities. Improving performance on core WM tasks should theoretically strengthen cognitive skills and lead to near and far transfer of performance gains. Due to the between subject nature of the task, we purposefully chose not to use adaptive tasks in nature as done in many cognitive training studies [24, 85]. Due to the between subject comparisons in performance gains, we made efforts to ensure that all tasks were equally difficult. Furthermore, no participants neared ceiling on any trained tasks indicating that participants were sufficiently challenged by each training task.

### Transfer tasks

#### Digit Span (near transfer)

This task measures short-term and WM capacity. Participants repeated a string of spoken numbers aloud as heard (forward) or in reverse order (backward). The number of digits increments by one digit until the participant failed two trials of the same length.

#### Stroop Task (far transfer)

This task measures selective attention. Color names are printed in congruent or incongruent ink colors, and participants press a button [1–7] that corresponds with the color of the ink, rather than the printed word. Participants initially completed practice trials to familiarize themselves with the correct response button. We instructed participants to answer as quickly and accurately as possible. Participants had unlimited response times. There were a total of 100 trials equally divided among congruent and incongruent trial types.

#### Spatial 2-back (near transfer)

This task measures WM performance. We instructed participants to remember the location of stimuli (green circles: 3° visual angle) appearing sequentially in one of nine locations (500 ms), followed by a blank delay (2000 ms). Participants pressed ‘j’ when the stimulus matched the location presented two trials previously; they pressed ‘f’ if the presented circle did not match. All participants completed at least 45 practice trials until they were comfortable with the timed task. The experimental task consisted of 138 trials (66% non-match) and lasted ~6 minutes.

### Trained Tasks

#### Visuospatial WM

**Fig 1:**
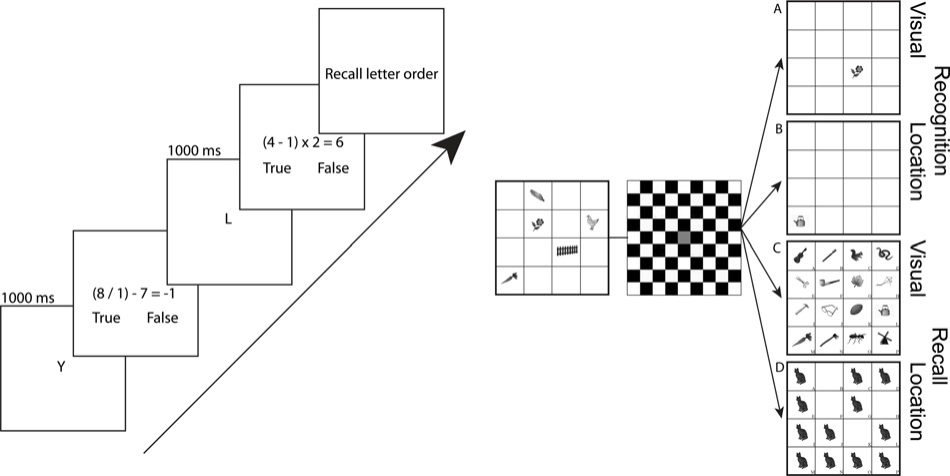
Left: OSpan WM paradigm. Participants remember consonants (1000 ms) then solve arithmetic problems before reporting the letter sequence. Right: Visuospatial WM paradigm. A) Visual recognition trials start with the presentation of the stimulus array (500 ms) followed by a delay period (750 ms) and the appearance of a probe item. Participants reported whether the probe item was ‘old’ or ‘new’. B) Location recognition trials began with the stimulus array (200 ms) followed by a delay period (4000 ms). Participants reported whether the probe location was ‘old’ or ‘new’. C) Visual recall trials begin with stimulus presentation (2000 ms) followed by a delay (500 ms). The probe array contained 15 new and 1 old item, which participants were asked to identify. D) Location recall trials begin with stimulus presentation (200 ms) followed by a delay period (4000 ms). At probe, an array of filled locations appeared and participants reported which filled location had been occupied at encoding.

These WM paradigms varied task demands (visual, spatial) and retrieval demands (recognition, recall); see Fig 1. The visual stimuli consisted of 20 grayscale drawings of common objects (e.g. cat, fence) [86]. Stimuli appeared in a 4x4 grid containing five items, followed by a delay interval filled with a checkerboard and a memory probe (unspeeded). The timing of individual tasks varied to try to equate performance across tasks based on pilot data. The four paradigms were presented separately in two pseudo-randomly ordered 25-trial blocks.

In the visual recognition task, five items were presented (500 ms), followed by a delay (750 ms), then one probe item returned, and participants made a new/old judgment, indicating whether or not the item was previously seen. In the spatial recognition task, five items were presented (200 ms) followed by a delay period filled with a checkerboard (4000 ms). Participants then decided if the returning item was in a new or old spatial location compared to the first five that were presented. In both the visual and spatial recognition trials, participants pressed the keys ‘o’ or ‘n’ to indicate whether the item or location was old or new, respectively.

In the visual recall task, five items were presented (2000 ms) followed by a delay period filled with a checkerboard (500 ms). 16 items then returned, filling each of the possible squares. Participants decided which 1 of the 16 items was present in the first 5. In the spatial recall task, five items were presented (200 ms) followed by a delay period filled with a checkerboard (4000 ms). Twelve images then returned each filled with a new picture, and only 1 of those 12 filled spatial locations was previously filled with the initial 5 items. In both the visual and spatial recall trials participants selected the correct item or location by selecting a letter (A-P) that corresponded with each of the 16 cells; see Fig 1.

#### The Automated Operation Span (OSpan)

This is a task of divided attention in which participants must solve arithmetic problems while simultaneously encoding and maintaining a list of letters [80]. Participants must recall letters after they complete the arithmetic problems. The task lasted ~10 minutes and consisted of nine sets of letters, which ranged from 3 to 7 total letters. We measured performance by letter recall and math accuracy (scores range from 0 to 50).

### TDCS Protocol

There were 4 tDCS groups: anodal PFC (F4 International 10–20 EEG System [87]), anodal PPC (P4), alternating anodal PFC and PPC, or sham stimulation (control). Participants were randomly assigned to a group and were blinded as to the tDCS protocol they received. The experimenters were aware of the stimulation protocol the participants received each session. The first site for the alternating PFC/PPC group was counterbalanced (i.e. PFC first or PPC first) across participants. Sham stimulation location was counterbalanced between PFC and PPC locations. There was no cathodal stimulation group, as we were only interested in improving performance and cathodal stimulation is generally linked to interruption of function. We also did not include a no-contact group that received only tDCS with no WM training, as previous research suggests that tDCS alone during rest exerts no effect on behavioral outcomes [61, 88].

Stimulation consisted of a single continuous direct current delivered by a battery-driven continuous stimulator (Eldith MagStim, GmbH, Ilmenau, Germany). Current (1.5 mA, 10 minutes) was delivered through two 5 x 7 cm^2^ electrodes housed in saline-soaked sponges. Sham stimulation included 20 seconds of ramping up and down at the beginning and end of stimulation to give the participant a physical sense of stimulation associated with current change. This effectively blinds participants to their stimulation condition [89]. Furthermore, no participants indicated that they believed that they were receiving sham stimulation, as tDCS was a novel research technique for all 72 participants. In all conditions, one electrode was placed over the target location at either F4 or P4 (International 10–20 EEG system) and the reference electrode was placed on the contralateral cheek. This reference location has previously been used effectively in tDCS studies of cognitive abilities [62, 63, 68, 69, 73, 90–92].

During the 10-minute stimulation/sham period, participants received task instructions and practiced all four visuospatial WM paradigms. Previous research shows that participating in cognitive tasks during tDCS benefits later performance [61]. After stimulation/sham, the electrodes were removed and the experimental trials began. TDCS effects last ~1 hour [40, 72, 93]; this study was designed to last less than an hour so that tDCS effects were present throughout testing, however some studies show shorter duration of tDCS effects [94, 95].

### Current Flow Modeling

To determine whether the tDCS stimulation was stimulating the frontoparietal networks central to WM performance, we modeled current flow. High-resolution models were derived from previous MRI data (1mm T2-weighted scan), not individually for participants in the current study. The MRI scans were segmented into several tissues: skin, fat, bone, CSF, gray matter, white matter, air, and deep brain structures. Segmentation was carried out using Simpleware ScanIP (Simpleware Ltd., Exeter, UK). The electrodes were created in SolidWorks (Dassault Systèmes Corp., Waltham, MA) and oriented on the head using ScanCAD (Simpleware Ltd., Exeter, UK). The head, now with the electrodes placed, was imported back to ScanIP to generate a volumetric mesh.

The meshes were then imported to a finite element solver, COMSOL Multiphysics 3.5 (COMSOL Inc., Burlington, MA). A model based on the data was created in the software's AC/DC module. Typical electrical conductivities (S/m) were assigned to each of the tissues and electrodes: Skin 0.465, Fat 0.025, Bone 0.01, CSF 1.65, Gray matter 0.276, White matter 0.126, Air 1e-15, Electrodes 5.99e7, Gel 0.3 [96]. Deep structures were treated as either white matter or gray matter. To simulate direct current stimulation, certain boundary conditions were applied. The surface of the anode was assigned a current density (-n **·** J = 1), the surface of the cathode was grounded (V = 0), internal boundaries were assigned continuity (n **·** (J_1_—J_2_) = 0), and the remaining surfaces were considered insulated (n **·** J = 0). The Laplace equation (V: potential, o: conductivity) was then solved [96]. After the simulation was run, the electric field magnitude was plotted on the surface of the gray matter.

## Results

### Current Modeling

**Fig 2:**
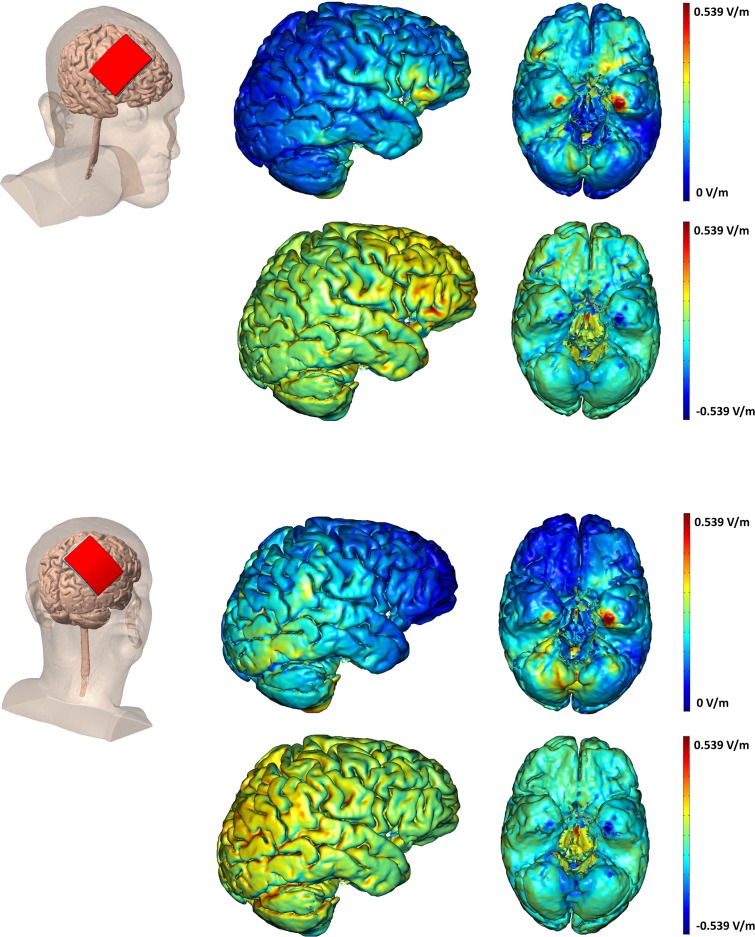
TDCS Current modeling. Modeling of current flow when applying 1.5 mA tDCS for F4 anodal (top) and P4 anodal (bottom) stimulation and the cathodal electrode placed on the contralateral cheek. The top row for each montage shows the electrical field (EF) magnitude plots. The bottom row for each montage is the radial EF plots showing the direction of stimulation. The red shows inward (anodal) EF, while blue represents outward (cathodal) EF.

We modeled current flow to more precisely identify the spatial extent of brain stimulation after anodal tDCS to PFC and PPC sites; see Fig 2. This analysis confirmed that tDCS to the PFC supplied current to PFC regions, but current also reached orbitofrontal and ventral temporal regions. Similarly, the PPC site stimulated PPC as well as more posterior occipital and ventral temporal regions. To our surprise, there was considerable overlap of current flow, suggesting that regardless of stimulation site, current reached frontoparietal networks strongly activated during WM performance.

### TDCS Effects

Based on the current modeling data showing overlapping current flow regardless of tDCS site, we first tested whether active tDCS predicted significant WM training and/or transfer benefits when compared to control (sham tDCS). To do this we created composite normalized difference scores termed benefit indices, by calculating normalized difference scores as follows: [(session 10 performance—session 1 performance)/(session 10 performance + session 1 performance)] for each participant and task. This normalization reduced variability across individuals’ performances and facilitated comparison across tasks with different scoring conventions. Furthermore, this comparison, which we previously employed in tDCS studies [62, 68, 97, 98], allows for analysis of improvement across all tasks, at each time point, which we then followed with individual analysis by task (see below). Composite indices were completed for the each of the 5 trained tasks and summing them to form the trained task benefit index, and separately for the 3 transfer tasks and summing those to form the transfer task benefit index. This provided us with a composite score for trained task improvement and a composite score for transfer task improvement. For the first analysis, performance in the control condition was compared to performance in the active groups, collapsing across the three active tDCS groups to form the ‘combined active’ group. We note that the same patterns emerged when raw measures of performance were used (for raw data see Table 1). Furthermore, performance on each trained and transfer task during session 1 was equivalent across all groups (Table 1).

**Table 1:** Accuracy/scores/reaction times for the five trained tasks and three transfer tasks.

	Sham			PFC			PPC			PFC/PPC		
Session	1	10	F.U.	1	10	F.U.	1	10	F.U.	1	10	F.U.
**Trained** Recog Visual	.73 (.07)	.77 (.10)	.75 (.10)	.73 (.07) [.79]	.75 (.06) [.57]	.75 (.08) [.97]	.73 (.06) [.78]	.78 (.07) [.75]	.76 (.07) [.82]	.72 (.09) [.78]	.74 (.09) [.80]	.73 (.08) [.51]
Recog Spatial	.73 (.14)	.80 (.13)	.78 (.10)	.72 (.14) [.88]	.79 (.14) [.86]	.75 (.12) [.53]	.73 (.16) [.98]	.80 (.15) [.98]	.81 (.12) [.39]	.73 (.14) [.96]	.81 (.14) [.73]	.85 (.09) [.02]
Recall Visual	.58 (.18)	.77 (.18)	.75 (.16)	.53 (.14) [.33]	.72 (.13) [.37]	.68 (.15) [.23]	.50 (.18) [.19]	.75 (.15) [.79]	.78 (.11) [.45]	.59 (.19) [.94]	.73 (.14) [.54]	.74 (.13) [.78]
Recall Spatial	.50 (.15)	.64 (.19)	.61 (.23)	.50 (.15) [.99]	.61 (.18) [.63]	.62 (.16) [.85]	.54 (.16) [.45]	.65 (.16) [.94]	.64 (.18) [.65]	.53 (.15) [.64]	.68 (.16) [.50]	.67 (.15) [.37]
OSpan	30.94 (9.52)	37.17 (6.32)	.34.72 (5.01)	25.39 (8.98) [.08]	35.06 (9.11) [.43]	34.22 (10.10) [.85]	31.17 (10.19) [.95]	34.78 (5.92) [.25]	36.61 (5.85) [.32]	28.72 (9.59) [.49]	33.72 (8.80) [.18]	36.28 (8.39) [.51]
**Transfer** Digit Span	13.0 (2.47)	12.78 (2.37)	13.17 (2.46)	12.56 (2.09) [.56]	12.33 (2.03) [.55]	12.89 (1.57) [.69]	12.3 (2.09) [.56]	12.83 (1.92) [.94]	12.67 (2.47) [.55]	12.6 (2.43) [.59]	12.56 (2.48) [.79]	13.06 (2.18) [.88]
Spatial 2-Back	.70 (.21)	.74 (.24)	.71 (.24)	.64 (.23) [.39]	.77 (.15) [.67]	.83 (.08) [.04]	.65 (.20) [.46]	.72 (.21) [.77]	.81 (.10) [.08]	.66 (.26) [.64]	.82 (.08) [.23]	.85 (.05) [.01]
Stroop	2140.3 (418.1)	2002.0 (401.9)	1993.8 (381.6)	2065.4 (444.9) [.61]	2057.4 (504.3) [.72]	2063.7 (485.9) [.63]	2495.3 (903.5) [.14]	2240.2 (554.1) [.15]	2225.6 (564.3) [.16]	2042.8 (424.4) [.49]	2012.3 (415.9) [.94]	1876.7 (334.2) [.33]

Paraenthesis represent standard deviation. Brackets represent t-test p value as compared to the sham group for the same task on the same session.

First, we tested the hypothesis that active tDCS promoted greater training and transfer gains as compared to control after 10 sessions of training. A repeated-measures ANOVA comparing the two cumulative benefit indices (trained, transfer) with the between-subjects factor of tDCS condition (combined active, control). After 10 sessions of training, there was a significant main effect of benefit index (trained, transfer) such that all participants had greater improvement on trained tasks (F_1, 70_ = 62.06, MSE = 3.45, p <.001, partial η^2^ <.47), this was expected, because there were more trained [5] than transfer tasks [3]. Importantly, there was no significant effect of tDCS condition (F_1, 70_ = .83, MSE = .02, p = .37, partial η^2^ = .01), and no interaction of tDCS group x benefit index (F_1, 70_ = .89, MSE = .05, p = .35, partial η^2^ = .01). To summarize, after ten sessions of WM training, both tDCS groups (active, control) showed equivalent improvement on trained and transfer tasks. Thus, WM training was effective and there were no differences as a function of group. In other words, by the end of the 10 sessions, active tDCS did not lead to significantly greater training gains or rate of training.

**Fig 3:**
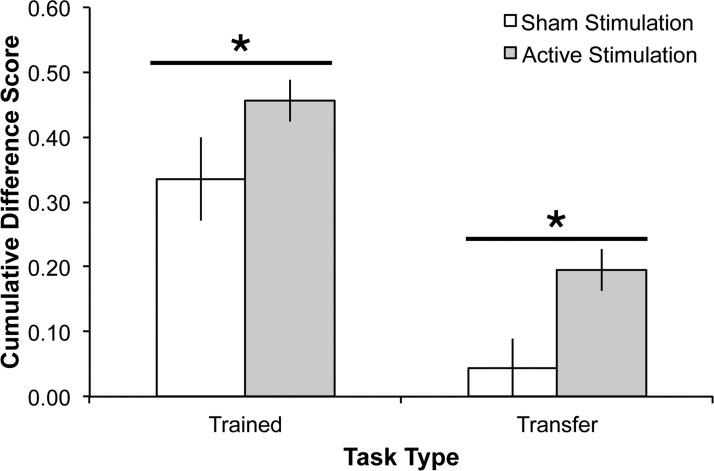
Combined benefit indices (follow-up compared to session 1) for the five trained and three transfer tasks for each stimulation group (active, sham). The active tDCS groups were collapsed across site because there was no significant difference between them. Error bars represent standard error of the mean.

However, at follow-up, after a month of no contact, a different pattern emerged. Repeating the analysis described above, using benefit indices from baseline incorporating follow-up performance, there was again a main effect of benefit index (F_1, 70_ = 34.54, MSE = 2.06, p <.001, partial η^2^ = .33), such that the trained benefit index was significantly greater than the transfer benefit index across both groups. Importantly, there was also a significant main effect of tDCS group (F_1, 70_ = 7.32, MSE = .25, p <.01, partial η^2^ = 0.10) such that the active tDCS group showed significantly greater performance across trained and transfer tasks; see Fig 3. This was driven largely by improvements on the more difficult spatial 2-back and OSpan tasks (see below). There was no group x benefit index interaction (F_1, 70_ = .10, MSE = .01, p = .75, partial η^2^ <.01). These findings demonstrate that active tDCS to frontoparietal sites sustained practice gains for trained WM tasks and enhanced transfer task performance. In other words, all groups showed practice related improvement, but only active tDCS sustained these gains.

A subsequent question arises as to how the different active stimulation groups performed compared to each other, as we grouped them together in the above analysis. To answer this, we tested the hypothesis that the different stimulation sites resulted in different training or transfer gains. We compared the two cumulative benefit indices (trained, transfer) across the three active tDCS groups (PFC, PPC, PFC/PPC) and found no main effect of site after session 10 (F_2, 51_ = .04, MSE <.01, p = .96, partial η^2^ <.01, all pairwise comparisons p >.80, or follow-up testing (F_2, 51_ = .30, MSE = .02, p = .74, partial η^2^ = .01, all pairwise comparisons p >.46). In other words, all active tDCS groups resulted in equivalent benefits regardless of stimulation site.

### Analysis by Task

**Fig 4:**
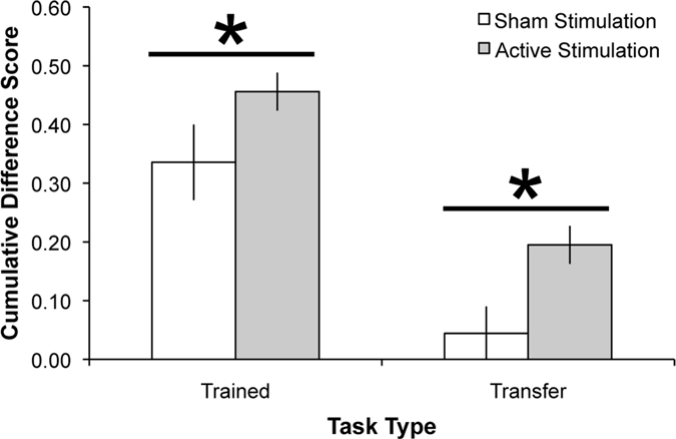
Performance gains per task. A: Stacked difference scores (follow-up compared to session 1) for the five trained tasks for the sham group and the average for the active stimulation groups. B: Stacked difference scores (follow-up compared to session 1) for the three transfer tasks for the sham group and the average for the active stimulation groups. Error bars represent standard error of the mean.

Lastly, we tested the hypothesis that the training and transfer gains for the active tDCS groups were disproportionally driven by individual tasks. A criticism would be that by grouping the tasks’ benefit indices together, we are hiding the individual effects tDCS has on each task. To investigate this possibility, we conducted a repeated measures ANOVA for the 5 trained task benefit indices from follow-up for the combined active tDCS group. There was a significant effect of task, (F_4, 204_ = 11.51, MSE = .19, p <.001, partial η^2^ = 0.18), such that the two recall tasks and the OSpan task provided significantly greater gains when compared to the two recognition tasks (recognition verbal compared to all other trained tasks: all p’s <.02, recognition spatial compared to all other trained tasks: all p’s <.04); see Fig 4. The only other significant difference was verbal recall provided significantly greater training gains than spatial recall (p = .03). There was no difference between the OSpan and either recall task (both p’s >.19). The task x group interaction was not significant (F_8, 204_ = 1.34, MSE = .02, p = .22, partial η^2^ = 0.05).

For the transfer tasks, the significant benefit was driven by the spatial 2-back task; see Fig 4. As above, there was a main effect of transfer task (F_2, 102_ = 14.95, MSE = .30, p <.001, partial η^2^ = 0.23), showing significantly greater improvement on the spatial 2-back task compared to the digit span and Stroop tasks (both p’s <.001). The pairwise comparison between the digit span and Stroop tasks was not significant (p = .45). The task x group interaction was not significant (F_4, 102_ = .28, MSE = .01, p = .89, partial η^2^ = 0.01). In sum, across trained and transfer tasks, the more challenging and adaptive tasks showed greater gains and transfer was observed for the near transfer task alone.

## Discussion

For many, maintaining cognitive performance is a priority in the aging process. Here, we confirmed the effectiveness of WM training paradigms and demonstrated that tDCS combined with WM training will lead to longer-lasting benefits in older adults. After ten sessions, all participants significantly improved across tasks. This was true regardless of whether the participant received active or sham tDCS. This finding is encouraging as it supports previous findings, which show the importance of WM training in enhancing or recovering cognitive skills in healthy older adults [13, 15, 23–37]. At follow-up, after one month of no contact, participants who received active tDCS performed significantly better on trained *and* transfer tasks than the control sham group. The magnitude of this effect did not vary as a function of stimulation site, PFC or PPC. In other words, tDCS-linked WM benefits emerged after training ended showing that tDCS helped maintain practice gains over time and enhanced transfer task performance. Thus, WM training when combined with tDCS offers promise in maintaining WM gains over longer periods of time. We offer that tDCS extends WM training benefits. This is especially relevant in a population concerned about their cognition: the healthy aging.

As noted, the current study provides convergent support for previous WM training studies reporting improved task performance after training [13, 15, 23–37]. In these studies, training benefits assess performance with measures that conflate practice effects with strengthened WM skills. Importantly, a recent meta-analysis of WM training studies found no difference in the training benefits associated with adaptive and nonadaptive training paradigms [99]. This report addresses a possible criticism of the current work in which nonadaptive training tasks were employed. We did observe the largest transfer effect in the most difficult near transfer task, the spatial 2-back WM. The two other near transfer measures, the Stroop task and the digit span showed no transfer effects. This is consistent with previous training studies, which report transfer for challenging WM tasks that require rapid updating (e.g. the n-back task; [29, 85, 100]).

The general benefit of tDCS holds promise in several domains. These data join four other studies showing that tDCS-linked cognitive training enhances performance across various domains. For example, six training sessions pairing the Stroop task with bilateral oppositional tDCS (left PFC anodal, right PFC cathodal) improved young adults’ performance [101]. Yet participants who received bilateral stimulation to the PPC (left anodal, right cathodal) showed improved numerical learning but impaired Stroop performance [101]. However, no follow-up measure was reported. A second study paired five training sessions with a related technique, transcranial random noise stimulation, to the PFC and found significantly enhanced arithmetic learning [102]. Next, one study paired tDCS with computer-assisted cognitive training in older adults [79]. They found that bilateral anodal tDCS to the PFC paired with verbal WM training improved trained task performance (verbal WM, digit span). Digit span improvements lasted seven days but they did not test transfer. Importantly, one other study found near transfer WM gains following 10 sessions of tDCS to the PFC in college aged students [103]. Our data replicate and extend these findings and show that longitudinal tDCS benefits WM over a potentially therapeutic time frame and it is appropriate for use in healthy older adults. Furthermore, these benefits were found following two different stimulation sites and persisted after one month of no contact.

Although tDCS shows promise for cognitive maintenance, the mechanism underlying long-term changes remains unclear. Previous evidence demonstrated temporary modulation of motor cortex [93], although long-term effects are reported to follow PFC stimulation [78, 104–106]. We targeted frontoparietal networks implicated in WM performance, and it appears that at least two, not exclusive, substrates could be contributing to the observed behavioral changes. One possibility is that tDCS strengthened the frontoparietal connections engaged during WM tasks. This may explain why there was no significant performance difference as a function of stimulation site. A second explanation for tDCS-linked WM benefits is that tDCS-linked WM training strengthened frontostriatal connections and enhanced striatal dopaminergic activity. This interpretation is based on work showing that frontostriatal activity is important for learning and WM updating (reviewed in [107]) and findings that WM training enhances striatal dopaminergic activity during WM updating in older [108] and younger adults [109], particularly in challenging WM tasks [110].

Although these findings demonstrate the feasibility and the durability of tDCS-linked WM training, there are several limitations to address in future investigations. A first question relates to the lack of spatial specificity afforded by the tDCS technique itself. As we found a benefit of tDCS at two different stimulation sites, we cannot rule out that tDCS to any portion of the cortex could lead to improved WM training gains. The PFC and PPC sites are both associated with WM performance and they were selected to enhance our likelihood of observing WM benefits. Future studies should include control locations expected to show no effect on WM, to clarify whether general stimulation promotes training gains. Given the exploratory nature of this first longitudinal study we elected to focus on training groups that seemed most likely to reveal improved performance rather than no change. Additionally, we cannot rule out those participants in the active tDCS groups showed placebo related benefits from the sensation of 1.5 mA tDCS. However, we believe the tDCS-naïve participants in the current study were unaware of the possibility of a sham condition as no participants indicated that they believed to be in a control group. Previous research finds that the tDCS sensation with 1.0 mA is not discernable from sham stimulation for naïve and experienced participants [111], however at 2.0 mA participants are able to detect the difference [112]. It is important to note however, that in those two studies participants received both active and sham tDCS in two different sessions, whereas in the current study participants received only active or sham tDCS.

A second parameter to optimize is the length of training. In the present manuscript, we used two weeks of WM training. However, one recent meta-analysis found that the *type* of training overshadowed the impact of training duration [99]. This was determined by a lack of a dose-response relationship between training length and near-transfer outcomes. Thus, the duration of training may be a secondary factor. Clearly, the shorter the training to achieve maximal benefits the better. Thirdly, this initial foray into tDCS paired with WM training revealed that performance benefits transferred. This is the most encouraging finding with regard to real-world application. Future work is now needed to clarify several aspects of transfer effects. New studies will need to include a stimulation control group that receives tDCS without WM training. This would clarify whether tDCS alone provided long-term benefits rather than the combination of tDCS+WM training. We think this is unlikely, because not every WM task benefits from tDCS (e.g. [68] easy WM tasks; [113]), and tDCS alone during rest exerts no effect on behavioral outcomes [61, 88], making a general, tDCS-induced long-lasting WM improvement unlikely. Additional work will be needed to ascertain the extent of transfer effects and to refine protocols to enable far transfer to other cognitive domains. Finally, with regard to transfer benefits, our transfer tasks were completed at three different time points in this study making them ‘trained’ to a certain degree. However, if the benefits really reflected training then we should have observed the same improvements in the sham group, which was not the case.

A final limitation is that the training tasks were computer-based. Future work should include tasks with greater ecological validity to clarify the translational power of tDCS in healthy aging and special aging populations. Furthermore, measures of far transfer will be needed to assess changes in remote cognitive domains such as fluid intelligence. Ideally, training will improve skills like sustained attention, that show transfer to daily skills like driving [114]. Improvement on cognitive functioning becomes important for maintaining autonomy and quality of life. Future work is needed to test longitudinal effects of tDCS in diverse tasks and in heterogeneous populations to predict who will benefit and for how long. However, the reality that neuroscience will be playing an important translational role has arrived. We offer this early work as encouragement to those of us engaged in the aging process and interested in maintaining cognitive function.

## Supporting Information

S1 Dataset (XLSX)
