# Correction: Longitudinal Neurostimulation in Older Adults Improves Working Memory

**DOI:** 10.1371/journal.pone.0129751

**Published:** 2015-05-29

**Authors:** Kevin T. Jones, Jaclyn A. Stephens, Mahtab Alam, Marom Bikson, Marian E. Berryhill

Fig 4 was incorrectly published as a duplicate of Fig 4. Please see the corrected Fig 4 here.

**Fig 4:**
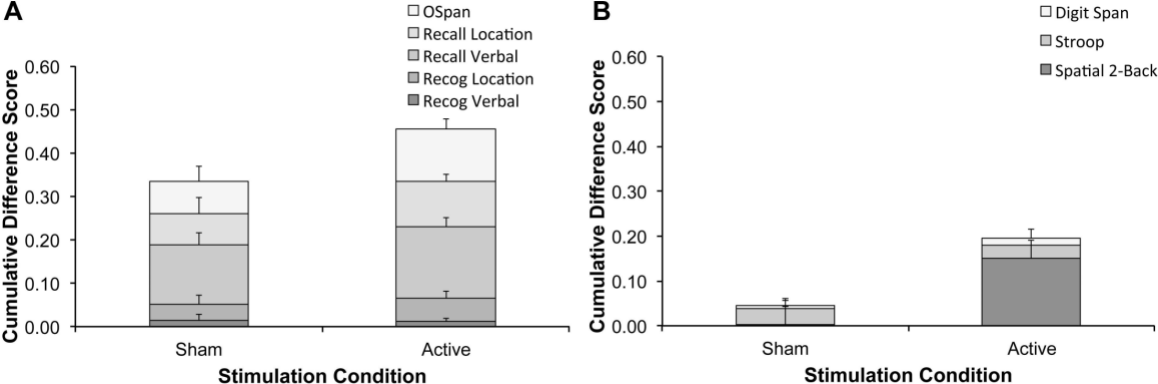
Performance gains per task. A: Stacked difference scores (follow-up compared to session 1) for the five trained tasks for the sham group and the average for the active stimulation groups. B: Stacked difference scores (follow-up compared to session 1) for the three transfer tasks for the sham group and the average for the active stimulation groups. Error bars represent standard error of the mean.
